# Placental basal plate with attached myofibers and adverse pregnancy outcomes: A systematic scoping review

**DOI:** 10.1111/aogs.70277

**Published:** 2026-06-09

**Authors:** Eric Jauniaux, Helena C. Bartels, Paul Downey, Francesco D'Antonio

**Affiliations:** ^1^ EGA Institute for Women's Health, Faculty of Population Health Sciences University College London (UCL) London UK; ^2^ Department of Obstetrics and Gynecology, Department of Pathology, National Maternity Hospital, School of Medicine University College Dublin Dublin Ireland; ^3^ Center for Fetal Care and High‐Risk Pregnancy, Department of Obstetrics and Gynecology University of Chieti Chieti Italy

**Keywords:** basal plate myofibers, histopathology, obstetric outcome, perinatal complications, placenta, placenta accreta

## Abstract

**Introduction:**

The finding of basal plate myometrial fibers (BPMF) delivered with the placenta has been associated with many different pregnancy and delivery complications. This study aims to evaluate the clinical utility of this histopathological finding in obstetrics.

**Material and Methods:**

We searched PubMed, Google Scholar, and Embase for studies published in English reporting an association between obstetric complications and placental BPMF, using combinations of relevant medical subject heading terms and keywords, published between March 1996 and December 2025. Study characteristics were evaluated by two independent reviewers using a predesigned protocol. The PRISMA Extension for Scoping Review (PRISMA‐ScR) was used to extract data and report the results.

**Results:**

Sixteen studies met our eligibility criteria for inclusion in this review. There were five cohorts and 11 studies, involving 5770 participants from four different countries. Nine studies reported on the association between BPMF on histopathologic examination in the setting of a delivered placenta and maternal or fetal pregnancy and delivery complications, and the remaining seven studies investigated the association between BPMF at birth in the index pregnancy and abnormal placental attachment at the next delivery. The most common prenatal and perinatal disorders were hypertensive disorders of pregnancy, placenta previa, preterm delivery, pre‐labor rupture of the membranes, suspicion of intrauterine infection, diabetes, stillbirth, and fetal growth restriction, and the most common intrapartum complications were placental abruption and placental retention. A higher incidence of abnormal placental attachment was reported in the subsequent pregnancy in cases where BPMF were found in the first pregnancy, but wide heterogeneity was observed across the included studies, particularly in the clinical criteria used to report placental attachment at delivery and interpretations of corresponding results.

**Conclusions:**

The clinical usefulness of reporting on BPMF in delivered placentas from pregnancies complicated by maternal or fetal disorders is currently difficult to evaluate due to variability in inclusion criteria, methodological protocols, and reported outcomes. The study of BPMF in delivered placentas may help fill evidence gaps in our knowledge of the pathophysiology of placental‐related pregnancy disorders involving the uteroplacental interface and the link between these disorders and long‐term maternal cardiovascular complications.

## Graphical abstract

The presence of basal plate myometrial fibers was associated with higher rates of placenta‐related pregnancy disorders and abnormal placental attachment in subsequent pregnancies, but heterogeneity in the data was found due to variable inclusion criteria, sampling methods, and reported outcomes.

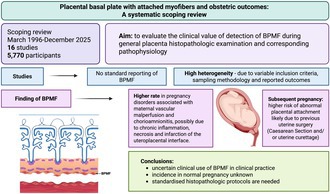

**Abbreviations**
BPMF: basal plate myometrial fibers
CD: cesarean delivery
FGR: fetal growth restriction
PAS: placenta accreta spectrum
PH: peripartum hysterectomy
PPH: postpartum hemorrhage

**Key message**

There is a lack of high‐quality evidence on the clinical significance of the histopathologic finding of basal plate myometrial fibers in delivered placentas in pregnancies complicated by placenta‐related disorders, particularly regarding its predictive value for abnormal placental adherence at the next delivery.

## 1 INTRODUCTION

**FIGURE 1:**
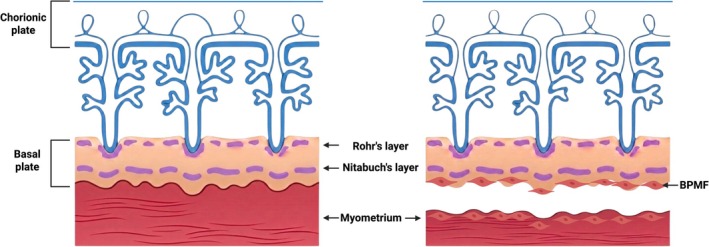
Diagram showing: Left. The normal layers of the placental basal plate before delivery; Right: After delivery with BPMF detached from the superficial myometrium.

The human placental basal plate is a layer of tissue that forms the maternal‐facing surface of the placenta and is primarily constituted of the decidua basalis, a decidualized endometrial tissue.^1^ Raissa Nitabuch^2^ was the first to describe the anatomy of the decidual layers and identify the spiral arteries in 1887. The basal plate of the definitive placenta comprises a complex mix of extravillous trophoblast and decidual cells enmeshed in fibrinoid material.^1^ The “Nitabuch membrane” is a continuous fibrinoid layer or stria that is laid down between the extravillous trophoblast of the cell columns of anchoring villi and the maternal decidual cells, which was identified as the site of detachment of the placenta from the uterine wall after the birth of the fetus.^2^ In addition to Nitabuch's layer, there is also Rohr's layer, which is a specialized zone of fibrinoid material located on the inner surface of the basal plate, positioned specifically beneath the cytotrophoblast shell and around the base of the anchoring villi (Figure 1).

An increase in the incidence of basal plate myometrial fibers (BPMF) delivered with the placenta was first reported by Sherer et al. in 1996 in a histologic examination of placentas from preterm births.^3^ The authors also found that placentas with BPMF weighed less and presented more frequently with the absence of the physiologic changes typically seen in the spiral arteries. The same year, Jacques al reported on a microscopic foci of myometrial tissue adherent to the basal plate with deficient intervening decidua in 36 delivered placentas described as mild or focal cases of placenta accreta.^4^ Several authors later developed the concept of focal‐occult accreta and its association with placenta accreta spectrum (PAS) at birth in subsequent pregnancies.^5^, ^6^, ^7^, ^8^, ^9^ The presence of BPMF has also been associated with retroplacental blood clots^10^ and preeclampsia.^11^

The physiological mechanisms underlying placental separation at delivery, as well as why myometrial fibers are attached to the basal plate in some cases but not in others, particularly in the context of various obstetric complications, remain uncertain. The aim of this study was to evaluate the clinical usefulness of reporting on BPMF during routine placental histopathology and discuss the possible mechanisms leading to the presence of BPMF in obstetric syndromes involving the uteroplacental interface.

## 2 MATERIAL AND METHODS

### 2.1 Eligibility criteria, information sources, and search strategy

We conducted searches in PubMed, Google Scholar, and Embase for studies published in English between the first description of an association between obstetric complications and placental BPMF at birth by Sherer et al. in March 1996^1^ and December 15, 2025. The search protocol was designed a priori and registered on PROSPERO (CRD420251069964) (www.crd.york.ac.uk/PROSPERO) in line with the Preferred Reporting Items for Systematic Reviews and Meta‐Analyses extension for Scoping Review (PRISMA‐ScR).^12^ We used MeSH headings, text words, and word variants for “basal plate myometrial fibers”, “basal plate myometrium”, “basal plate myofibers”, and “obstetric outcome”, “perinatal complications”, “placenta accreta”, “placenta increta”, “abnormally invasive placenta”, “morbidly adherent placenta”, “occult placenta accreta”. We combined these MeSH headings, text words, and word variants with terms related to “maternal serum”, “serum biomarkers”, and “prenatal diagnosis”. The systematic search was supplemented with a hand search of Google Scholar and other sources, including conference and meeting abstracts and proceedings, as well as the reference lists of reviews and editorials.

### 2.2 Article selection

Two independent investigators (EJ and HB) screened titles and abstracts of all citations for potentially relevant papers and selected the studies in two stages: first, the abstracts of all potentially relevant articles were individually examined for suitability; secondly, the remainder were obtained in full text and independently assessed for content, data extraction, and analysis. Additional relevant studies were identified from the reference lists of reviews and editorials. Disagreements between the two original reviewers were resolved by discussion with the third investigator (FA).

We included observational studies (cohort or case–control) reporting on the presence of BPMF during the histopathologic examination of the placenta. We excluded case reports, case series of <10 cases, letters, and reviews.

### 2.3 Data extraction

Clinical study characteristics were subsequently extracted independently by two reviewers (EJ and HB) using a predesigned data extraction form, which included the following information for all studies: study design, study type, sample size, recruitment setting, year of publication, country of origin, methodology, and outcomes.

### 2.4 Data synthesis

The evaluation of the data identified in the present review was limited by wide variations in methodology and by the availability of clinical data for specific placental‐related complications of pregnancy and delivery. Our study, initially designed as a systematic review and meta‐analysis, was subsequently redesigned as a systematic scoping study.

## 3 RESULTS

**FIGURE 2:**
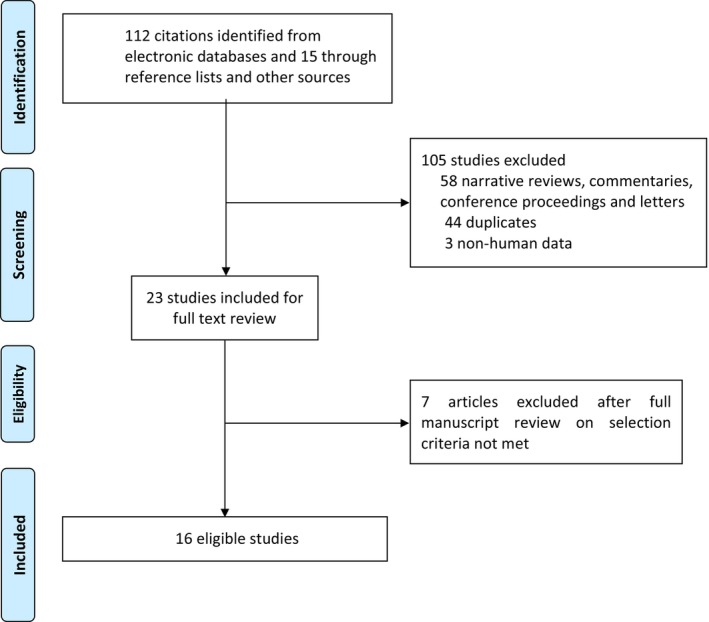
Flow diagram showing the selection of articles included in the review.

Out of the 127 citations identified through the literature search, 16 studies^3^, ^4^, ^5^, ^6^, ^7^, ^8^, ^9^, ^10^, ^11^, ^13^, ^14^, ^15^, ^16^, ^17^, ^18^, ^19^ met our eligibility criteria for inclusion in this review and are reported in the PRISMA flow diagram (Figure 2).

### 3.1 Study characteristics

Characteristics of the included studies are presented in Table 1. There were five cohorts and 11 studies, involving 5770 participants from four different countries. Fourteen studies were retrospective, and two were prospective, including one case–control study evaluating the presence of BPMF in placentas from term deliveries complicated by placental retention^9^ and one cohort study evaluating placentas presenting with retroplacental blood clot.^10^ In all 11 case–control studies, the cases and controls were obtained from the same pathology laboratory but were unmatched for maternal characteristics, labor complications, and mode of delivery. Only one study provided indications for placental histopathologic examination in both cases and controls.^8^

**TABLE 1:** Main characteristics of the 15 studies included in the systematic review.

Author, year [Reference]	Country	Study period	Type of study	Study design and number of patients in subgroups	History of uterine surgery and evidence of BPMF on prior placenta
Sherer et al^3^ 1996	USA	1988–1994	Retrospective	Case–control 457 premature births <32 weeks vs. 108 (unmatched) uncomplicated term singleton births.	Not reported
Jacques et al^4^ 1996	USA	1993–1994	Retrospective	Cohort 36 placentas with BPMF out of 1500 placentas examined for maternal or fetal complications, including premature delivery, suspected infection, placenta accreta, and stillbirth.	BPMF (*n* = 36) 23 D&C/10 CD/3 D&C + CD
Khong et al^5^ 2001	USA	1999–2000	Retrospective	Cohort 90 placentas, including 26 with BPMF	BPMF (*n* = 26): 3 D&C/3 CD vs. No BPMF (*n* = 64): 3 D&C/11 CD
Stanek et al^6^ 2007	USA	ND	Retrospective	Case–control 25 placentas with BPMF vs. 25 without BPMF (unmatched)	BPMF (*n* = 25): 1 CD vs. controls (*n* = 25): 1 CD
Linn et al^7^ 2015	USA	2008–2013	Retrospective	Case–control 150 placentas from consecutive pregnancies, including 50 diagnosed with clinical suspicion and/or pathologic diagnosis of placenta accreta in the second pregnancy vs. 100 controls with no evidence of placenta accreta in the index and prior pregnancy (unmatched)	MAP (*n* = 50): 25 D&C^a^/24 CD/23 MROP vs. controls (*n* = 100) 11 D&C/47 CD/15 MROP BPMF^a^: MAP = 42 (84%) vs. controls = 42 (42%)
Miller et al^8^ 2015	USA	2008–2013	Retrospective	Case–control 125 cases with at least two consecutive pregnancies with placental histopathology, including 25 diagnosed clinically and pathologically with MAP vs. 100 controls with no evidence of placenta accreta in index and prior pregnancy (unmatched)	MAP (*n* = 25) 12 D&C/15 CD vs. controls (*n* = 100) 11 D&C/48 CD BPMF^a^: MAP = 19 (76%) vs. controls = 41 (41%)
Endler et al^9^ 2016	Sweden	2013–2014	Prospective	Case–control 100 live term vaginal birth, including 50 cases with placental retention vs. 50 controls without retention from live term singleton pregnancies Placenta accreta was defined as a focal absence of intervening decidua between the chorionic plate and the myometrium.	Retained placenta (*n* = 49): 11 D&C/2 CD vs. controls (*n* = 47): 14 D&C/2 CD
Wyand et al^10^ 2017	USA	June‐Oct 2016	Prospective	Cohort 156 routine placentas with retroplacental blood clots, including 21 with BPMF	4 CD
Wang et al^11^ 2018	USA	2009–2016	Retrospective	Case–control 230 complicated by hypertensive disorders of pregnancy vs. 76 controls with a history of malignancy and no hypertension (unmatched)	Hypertensive disorders cases (*n* = 230): 29 D&C/13 CD vs. controls (*n* = 76): 13 D&C/9 CD
Heller et al^13^ 2019	USA	2009–2013	Retrospective	Case–control 185 consecutive pregnancies with previous placental histopathologic examination, including 135 placentas with BPMF vs. 50 without BPMF (unmatched)	Not reported
Sato et al^14^ 2019	Japan	2004–2017	Retrospective	Case–control 84 cases of MAP/placenta accreta defined as villi adjacent to the myometrium without decidua with BPMF on histologic examination vs. 51 controls with no evidence of placenta accreta (unmatched)	MAP/Accreta (*n* = 84) 23 D&C^a^/34 CD^a^ vs. controls (*n* = 51) 14 D&C/7 CD
Thakur et al^15^ 2022	India	ND	Retrospective	Case–control 100 placentas from low‐risk term pregnancies, including 50 from VB and 50 from CD (unmatched)	Not reported
Stanek^16^ 2023	USA	2007–2020	Retrospective	Case–control 907 placentas from VB and 830 from consecutive CD investigated for mild forms of placenta creta (occult placenta accreta) as diagnosed by the presence of BPMF in the decidua (unmatched)	Not reported
Ravikumar et al^17^ 2024	India	2018–2020	Retrospective	Cohort 538 placentas from preterm livebirth (26–37 weeks)	Not reported
Erfani et al^18^ 2024	USA	2012–2020	Retrospective	Case–control 1344 consecutive pregnancies with previous placental histopathologic examination, including 119 with BPMF vs. 1231 controls without BPMF (unmatched)	BPMF (*n* = 119) 13 CD vs. controls (*n* = 1231) 184 CD
Hecht et al^19^ 2025	USA	2016–2020	Retrospective	Cohort 121 consecutive pregnancies with BPMF (Stage 2)	BPMF (*n* = 121) 4 CD (2+)/6 myomectomy/

Abbreviations: CD, cesarean delivery; MAP, morbidly adherent placenta; ND, not described; PIH, Pregnancy‐induced hypertension; VB, vaginal birth.

^a^ Statistically significant difference.

Nine studies reported on the association between the presence of BPMF on placental histopathologic examination and maternal or fetal pregnancy and delivery complications,^3^, ^4^, ^5^, ^6^, ^9^, ^10^, ^11^, ^15^, ^17^ and the remaining seven studies^7^, ^8^, ^13^, ^14^, ^16^, ^18^, ^19^ specifically investigated the association between BPMF at examination of the placenta performed for various pregnancy and perinatal complications at the first delivery and abnormal placental attachment described as morbidly adherent placenta,^7^, ^8^, ^14^ placenta accreta,^13^, ^18^, ^19^ or occult placenta accreta^16^ at the next delivery. Among the latter studies, three designed their study groups or cohort to include cases with clinical suspicion and/or pathologic diagnosis of placenta accreta^7^, ^8^, ^18^ with no description of the clinical criteria used by the obstetrical team, three^13^, ^14^, ^19^ reported as suspicion of morbid adherence or accreta placentation those presenting with retained placenta, manual removal, fragmented placenta, uterine curettage for partial placental retention, ultrasound evidence of accreta, adherent placenta and/or fetal membranes detected at delivery, and torn cotyledons during placental removal. Two studies^9^, ^16^ referred to the 1937 histologic description proposed by Irving and Hertig,^20^ i.e., absence of decidua between the placental villi and uterine myometrium, to confirm the diagnosis of mild or focal accreta (occult). In addition to the clinical criteria for morbid adherence, one study^14^ reported using the presence of villi adjacent to the myometrium, without decidua, as the diagnostic criterion for placenta accreta, increta, and percreta.

Nine studies^3^, ^4^, ^7^, ^9^, ^10^, ^16^, ^17^, ^18^, ^19^ described the use of a standard protocol for placental examination after formalin fixation, which involves sampling the cord, free membranes, the full thickness of the placenta (including the basal plate), and macroscopic lesions (Table S1). In six studies, the authors used immunohistochemistry in addition to routine hematoxylin and eosin (H&E) staining to identify BPFM,^4^, ^5^, ^6^, ^10^, ^11^, ^15^ and one reported on the use of an image analysis system to quantify the BPMF areas.^14^

### 3.2 Association between BPMF and pregnancy outcomes

All studies included placentas from pregnancies with different prenatal disorders or presenting with peripartum complications (Table 2). The most common prenatal and perinatal disorders were hypertensive disorders (preeclampsia, pregnancy induced hypertension, chronic hypertension), placenta previa, preterm delivery, pre‐labor rupture of the membranes, suspicion of intrauterine infection (chorioamnionitis, placental inflammation), diabetes (preexisting diabetes and gestational diabetes mellitus), stillbirth and fetal growth restriction (FGR) and the most common intrapartum complications were placental abruption and placental retention. Three case–control studies reported a statistically significant difference between cases and controls for chorioamnionitis,^6^ diabetes,^11^ stillbirth,^16^ and preeclampsia.^16^

**TABLE 2:** Clinical and Outcome data of the studies included in the systematic review.

Author, year (Reference)	Mode of delivery	Pregnancy and obstetric complications associated with BPMF on histopathologic examination
Sherer et al^3^ 1996	BPMF (*n* = 44): 33 CD vs. controls (*n* = 413) 263 CD	BPMF vs. controls: PROM *n* = 21 vs. *n* = 171; preeclampsia *n* = 9 vs. *n* = 67; PTB *n* = 9 vs. *n* = 149; abruption *n* = 4 vs. *n* = 27
Jacques et al^4^ 1996	21 CD out of 36 cases with BPMF	Cohort (*n* = 36): Delayed placental separation with MROP (*n* = 15); diagnosed clinically with placenta accreta & PPR (*n* = 4)
Khong et al^5^ 2001	BPMF 5 CD (non‐BPMF 11 CD)	BPMF cases (*n* = 26): Prematurity (*n* = 18); PROM (*n* = 7); abruption (*n* = 7); preeclampsia (*n* = 18); FGR (*n* = 9); preeclampsia & FGR (*n* = 4); fetal distress (*n* = 11); suspected infection (*n* = 4); and others (*n* = 12); placenta previa (*n* = 3); MROP (*n* = 3)
Stanek et al^6^ 2007	BPMF 14 CD vs. controls 11 CD	BPMF vs. controls: chorioamnionitis *n* = 6 vs. *n* = 16^a^; hypertension *n* = 3 vs. none; GDM *n* = 3 vs. *n* = 2; placenta previa *n* = 2 vs. none; abruption *n* = 1 vs. *n* = 1. No hysterectomies, PPH, or delayed placental separation with MROP in either group
Linn et al^7^ 2015	MAP 32 CD vs. controls 42 CD	MAP vs. controls: hypertension *n* = 3 vs. *n* = 9; preeclampsia *n* = 1 vs. *n* = 10; GDM *n* = 4 vs. *n* = 15; MROP *n* = 26 vs. *n* = 14; PH *n* = 15 vs. none^a^
Miller et al^8^ 2015	Not reported	MAP vs. controls: stillborn none vs. *n* = 3; FGR *n* = 1 vs. *n* = 6; preeclampsia *n* = 1 vs. *n* = 10; PTB *n* = 4 vs. *n* = 11; PROM none vs. *n* = 16; chorioamnionitis *n* = 1 vs. *n* = 7; abruption none vs. *n* = 2; fetal distress *n* = 4 vs. *n* = 10; placenta previa *n* = 11 vs. *n* = 3; PH^a^ *n* = 11 vs. none
Endler et al^9^ 2016	All VB	Retained placenta (*n* = 50) vs. controls (*n* = 50): BPMF *n* = 11 vs. *n* = 2^a^; focal accreta *n* = 6 vs. *n* = 2; maternal underperfusion *n* = 24 vs. *n* = 13; placental inflammation *n* = 20 vs. *n* = 22
Wyand et al^10^ 2017	Not reported	BPMF cases (*n* = 21):GDM (*n* = 6); hypertension (*n* = 7); preeclampsia (*n* = 2); PTB (*n* = 1)
Wang et al^11^ 2018	Cases 104 CD vs. controls 19 CD	Cases vs. controls: diabetes *n* = 24 vs. *n* = none^a^; autoimmune disorder *n* = 9 vs. *n* = 3; PTB *n* = 17 vs. *n* = 1; placenta previa *n* = 2 vs. *n* = none; abruption *n* = 3 vs. none; FGR *n* = 10 vs. none; MROP *n* = 29 vs. *n* = 5. BPMF *n* = 76 vs. *n* = 23^a^
Heller et al^13^ 2019	Not reported	PH in successive pregnancies for placenta increta and recurrent BPMF (*n* = 5); progression from BPMF to MAP (*n* = 5)
Sato et al^14^ 2019	MAP 57 CD vs. controls 33 CD	MAP cases (PAV;PI;PP) vs. controls (BPMF *n* = 54 vs. *n* = 7^a^): Placenta previa *n* = 8/*n* = 10/*n* = 12 vs. *n* = 5; PH *n* = 8/*n* = 15/*n* = 14 vs. none^a^
Thakur et al^15^ 2022	50 CD vs. 50 VB	All uncomplicated pregnancies (CD vs. VB): BPMF *n* = 39 vs. *n* = 40
Stanek^16^ 2023	830 CD vs. 907 VB	CD vs. VB: diabetes *n* = 60 vs. *n* = 47; hypertension *n* = 56 vs. *n* = 61; preeclampsia *n* = 112 vs. *n* = 50^a^; PROM *n* = 107 vs. *n* = 140; APH *n* = 101 vs. *n* = 98; stillbirth *n* = 40 vs. *n* = 236^a^; FGR *n* = 157 vs. 164; PPH *n* = 57 vs. *n* = 78; PH *n* = 5 vs. *n* = 1. Placenta accreta: *n* = 89 vs. *n* = 78
Ravikumar et al^17^ 2024	387 CD & 151 VB	All PTB with additional complications: PPROM (*n* = 121); APH (*n* = 56); preeclampsia or eclampsia or chronic hypertension (*n* = 251), FGR (*n* = 99), GDM (*n* = 55). BPMF *n* = 47
Erfani et al^18^ 2024	BPMF 62 CD vs. controls 683 CD	BPMF vs. controls: PH *n* = 3 vs. *n* = 12; placenta previa *n* = 1 vs. *n* = 15; PAS *n* = 3 vs. *n* = 3^a^
Hecht et al^19^ 2025	58 CD & 63 VB	FIGO I (MROP with curettage at VB or PPH with suturing of the placental bed at CD) *n* = 25 vs. FIGO 0 (no complications) *n* = 96. MROP *n* = 11 vs. *n* = 37; D&C or suturing *n* = 24 vs. *n* = 0^a^. Placenta previa *n* = 14 (*n* = 2 vs. *n* = 12)

Abbreviations: APH, antepartum hemorrhage; CD, cesarean delivery; D&C, dilatation & curettage; FIGO, International Federation of Gynecology and Obstetrics; GDM, gestational diabetes mellitus; MAP, morbidly adherent placenta; MROP, manual removal of the placenta; PAS, placenta accreta spectrum; PAV, placenta accreta vera; PH, peripartum hysterectomy; PI, placenta increta; PP, placenta percreta; PPH, post‐partum hemorrhage; PPR, partial placenta retention; PROM, premature rupture of the membranes; PTB, preterm birth; VB, vaginal birth.

^a^ Statistically significant difference.

Studies investigating the placenta in consecutive pregnancies^7^, ^8^, ^13^, ^18^, ^19^ reported a significantly higher incidence of abnormal placental adherence (morbidly adherent placenta, retained placenta) in cases presenting with BPMF in the first pregnancy. One of these studies used the following clinical criteria to include cases as PAS in their cohort: delivery requiring manual extraction, the need for a sharp curette to remove retained placental tissue, postpartum hemorrhage requiring oversewing of the placental bed, or another surgical procedure.^19^ Two studies reported a significantly higher incidence of prior dilatation and curettage (D&C)^7^, ^14^ and prior cesarean delivery (CD)^14^ in patients with abnormal placental attachment and BPMF. Three studies reported a significantly higher rate of peripartum hysterectomy (PH) in patients clinically diagnosed with abnormal placental attachment.^7^, ^8^, ^14^, ^18^

## 4 DISCUSSION

Our scoping review identified five cohorts and 11 case–control studies (Table 1) that evaluated the association between the presence of BPMF on pathologic examination of delivered placenta and pregnancy outcomes (Table 2). Based on our review, we identified two types of studies that associate BPMF with pregnancy and obstetric complications: firstly studies that describe the presence of BPMF in pregnancies complicated by various maternal disorders and delivery complications,^3^, ^4^, ^5^, ^6^, ^9^, ^10^, ^11^, ^15^, ^17^ and secondly studies that report a specific association between BPMF at examination of the placenta in the first pregnancy and abnormal placental attachment at birth in the same patient in the subsequent delivery.^7^, ^8^, ^13^, ^14^, ^16^, ^18^, ^19^ Overall, we identified substantial variability in the evidence regarding the clinical protocols used to identify and report BPMF incidence in both normal and complicated pregnancies, which limits interpretation of BPMF's clinical significance in the delivered placenta. In particular, key items are lacking, including quantification and distribution of BPMF in the placenta across different pregnancy and delivery complications, as well as a standardized clinical definition for abnormal placental adherence.

Microscopic fragments of the myometrium are frequently found in curettage specimens from the termination of first‐trimester pregnancies or the evacuation of retained products of conception in missed or incomplete miscarriage.^21^ Uterine curettage typically involves dilating the cervix, followed by evacuation of the uterine content using suction aspiration or curettage with a metal curette. Therefore, it is not surprising that myofibres would be present in those specimens. Curettage may also rarely cause permanent damage to the inner layer of the myometrium and perforation of the full‐thickness of the uterine wall.^21^, ^22^ Like hysterotomy procedures for CD, these surgical complications are associated with permanent damage to the uterine wall integrity and known to be associated with a higher incidence of placenta accreta spectrum (PAS) in subsequent pregnancies.^23^

The mechanisms leading to the presence of BPMF in the delivered placenta, including different pregnancy complications, have not been investigated. Maternal vascular malperfusion or uteroplacental insufficiency in placental‐related disorders of pregnancy, such as preeclampsia and FGR or chronic maternal hypertension in type I diabetes, for example, is the consequence of the failure of the extravillous trophoblastic migration and remodeling of the spiral arteries in early pregnancy.^24^, ^25^ Insufficient uterine vascular remodeling leads to abnormal placental perfusion, which reduces placental growth, contributes to chronic oxidative stress of the placental tissue, and increases the risk of premature uteroplacental separation.^26^, ^27^ Histopathologic studies have shown that maternal vascular malperfusion associated with preeclampsia and FGR is linked to an increased incidence of decidual arteriopathy, villous infarction, massive perivillous fibrin deposition, maternal floor infarction, and placental abruption.^27^, ^28^ Increased fibrin deposition has also been found in gestational diabetes mellitus GDM and stillbirth, and maternal vascular malperfusion lesions are often associated with inflammatory lesions in these cases.^24^, ^29^, ^30^ Chorioamnionitis, which is usually due to bacterial or fungal infections ascending from the cervicovaginal tract, has also been reported in association with BPMF (Table 2). Overall, these data suggest that chronic inflammation, necrosis, and infarction of the uteroplacental interface are likely to be involved in the mechanisms underlying myofiber detachment from the uterine myometrium at delivery (Figure 1). Prospective studies comparing the distribution of BPMF by clinical characteristics and pregnancy outcomes, such as preeclampsia,^25^, ^28^, ^31^ could also be useful for better understanding the mechanisms leading to the development of secondary complications such as FGR or placental abruption, and long‐term maternal cardiovascular disorders.^32^, ^33^

The association between BPMF and PAS is more complex to evaluate. In seven studies included in the present review, the authors used descriptions such as manual removal of the placenta with torn cotyledons, adherent placenta and/or fetal membranes with difficult placental delivery resulting in fragmentation of the placenta requiring uterine curettage for partial placental retention, to report on the association between BPMF and PAS.^7^, ^8^, ^13^, ^14^, ^16^, ^18^, ^19^ These descriptions are subjective and depend on the obstetrician's experience.^34^ It is therefore not surprising that the placental samples obtained from uterine curettage in these studies show an increased incidence of BPMF. When Irving and Hertig published the first cohort on placenta accreta in 1937, most of their cases were described as superficial accreta (creta or vera) and involved mainly the upper uterine segment.^20^ They hypothesized that the pathological basis for accreta placentation was the complete or partial absence of the decidua basalis with direct attachment of the villous tissue to the superficial myometrium or adjacent to myometrial fibers. However, the visualization of placental villi directly attached to the myometrium requires access to samples from hysterectomy or partial myometrial resection specimens, which is not available when the placenta can be fully detached in whole or in part from the uterine wall after manual removal, as in most of the cases included in the studies in the present review.

Six studies included in the present review^7^, ^8^, ^13^, ^16^, ^18^, ^19^ investigated the placenta from consecutive pregnancies to evaluate the association between BPMF in index pregnancies and abnormal placental attachment at the next delivery. All of the placentas in the index pregnancy were referred for histopathologic examination following a pregnancy complication such as preeclampsia, FGR, maternal infection, or an obstetric suspected abruption, such as non‐reassuring fetal heart and suspected abruption as recommended by the US guidelines for placental pathologic examination.^35^ These conditions were found to be associated with a high incidence of BPFM in the other studies included in the present review, highlighting a major bias in the selection of cases in those studies that linked BPMF in the index pregnancy to PAS in the next pregnancy. Furthermore, most of the clinical indications for placental pathologic examination in the index pregnancy often require urgent delivery by CD, and the significantly higher rate of peripartum hysterectomy (PH) in patients in the next pregnancy^7^, ^8^, ^18^ suggest that the factor leading to PAS in those cases was placentation in a surgical scar i.e., cesarean or curettage, rather than simply the presence of BPMF at the first delivery.

Our scoping review has both strengths and limitations. We performed comprehensive literature searches for all studies reporting on BPMF, pregnancy disorders, and perinatal complications, making it unlikely that we missed any significant publications. All studies included in our systematic review reported that BPMF was diagnosed by perinatal pathologists on microscopic examination. The main limitation of our study was the high heterogeneity across study designs, which precluded a meta‐analysis. We identify several other methodological issues with the article included in the present scoping review. Most studies were retrospective, and pathologists must rely on clinical information provided by the obstetric team at birth, which is often limited in studies that collect cases over long periods (Table 1).

Clinical indications for placental pathologic examination vary between clinicians, institutions, and countries, introducing a major bias in the selection of cases and controls. Pathologic examination may yield a false‐negative result when there is abnormal placental attachment, because the adherent area may not be delivered with the placenta and may not be examinable. Clinicians may also use different definitions for the different types of hypertensive disorders of pregnancy and for FGR, mixing cases of fetuses constitutionally small (small‐for‐gestation‐age) and/or born prematurely following inaccurate gestational dating or unknown gestational age (low birth weight). Even though most studies on BPMF originate in the USA, clinical definitions may also vary across institutions and healthcare systems over time. In addition to these selection biases, methodological disparities, such as the location and number of samples used for histopathologic examination, make it challenging to correlate clinical data with histopathologic findings across many studies.

## 5 CONCLUSION

The study of BPMF in delivered placentas may help fill evidence gaps in our knowledge of the pathophysiology of placental‐related pregnancy disorders involving the uteroplacental interface and the link between these disorders and long‐term maternal cardiovascular complications. The clinical usefulness of finding BPMF at birth on future pregnancies is currently difficult to evaluate due to wide heterogeneity in the data, particularly in the context of PAS, as the link between BPMF and the various clinical criteria used to describe complications of placental delivery has been overstated in the literature and overemphasized in clinical practice. There is a need for prospective studies quantifying the incidence and distribution of BPMF in normal pregnancies and deliveries, as well as for standardized protocols for collecting clinical data and sampling the placenta in complicated pregnancies and deliveries.

## AUTHOR CONTRIBUTIONS

**Eric Jauniaux:** Writing—review and editing, conceptualization, methodology, investigation, formal analysis, data curation, investigation, visualization. **Helena Bartels:** Writing—review and editing, data curation conceptualization, investigation. **Paul Downey:** Writing—review and editing, conceptualization. **Francesco D'Antonio:** Writing—review and editing, methodology, visualization, conceptualization.

## FUNDING INFORMATION

No external funding was provided for this study.

## CONFLICT OF INTEREST STATEMENT

The authors declare that they have no known competing financial interests or personal relationships that could have appeared to influence the work reported in this paper.

## Supporting information


Table S1.

## Data Availability

The data that support the findings of this study are available from the corresponding author upon reasonable request.
