# Supplementary material for: Placental basal plate with attached myofibers and adverse pregnancy outcomes: A systematic scoping review
Source: Acta Obstet Gynecol Scand. 2026 Jun 9;105(8):1435–44. doi: 10.1111/aogs.70277 (PMC13356478; doi:10.1111/aogs.70277)
Supplement: Supplementary file 1 — Table S1. [file AOGS-105-1435-s001.docx]

**Supplementary Table 1:** Description of the histopathologic protocol used in the different studies

| **Author**  **Year**  **[Reference]** | **Histopathologic examination** |
| --- | --- |
| Sherer et al^3^  1996 | Standard protocol: samples from UC (n=2), membranes (n=2)/ full placental thickness (n=4) & gross lesions.  H&E |
| Jacques et al^4^  1996 | Standard protocol: samples from UC (n=2), membranes (n=1)/ full placental thickness (n=4).  H&E & IHC |
| Khong et al^5^  2001 | Samples from intact (1 per 2cm area), disrupted and mixed areas of BP.  H&E & IHC. |
| Stanek et al^6^  2007 | Archive slide review.  H&E & IHC. |
| Linn et al^7^  2015 | Standard protocol for both pregnancies: samples of full placental thickness (n=2), BP biopsies along the long axe of the maternal surface (n=1) & gross lesions. H&E. |
| Miller et al^8^  2015 | Archive slide review.  All study cases had BP samples for histologic examination.  H&E. |
| Endler et al^9^  2016 | Standard protocol: samples from UC (n=1); full placental thickness (n=3) & gross lesions.  H&E. |
| Wyand et al^10^  2017 | Standard protocol: samples from UC (n=1), membranes (n=1)/ full placental thickness (n=2) & gross lesions.  H&E & IHC. |
| Wang et al^11^  2018 | Archive slide review  H&E |
| Heller et al^13^  2019 | H&E & IHC. |
| Sato et al^14^  2019 | 4-6 samples of full placental thickness with intact BP at random. The BPMF areas were quantified using image analysis.  H&E |
| Thakur et al^15^  2022 | Samples of full placental thickness (n=2), BP biopsies from the maternal surface (n=5).  H&E & IHC. |
| Stanek^16^  2023 | Samples with BPMF in the decidua or in direct contact with the Rohr fibrinoid or chorionic villi.  H&E. |
| Ravikumar et al^17^ 2024 | Standard protocol: samples from UC (n=2), membranes (n=1)/ full placental thickness (n=3).  H&E. |
| Erfani et al^18^  2024 | Standard protocol: samples from UC (n=2), membranes (n=1)/ full placental thickness (n=2).  H&E. |
| Hecht et al^19^ 2025 | Standard protocol: random samples full placental thickness (n=3) and from gross lesions (n=2). |

BP= Basal plate; H&E= Hematoxylin & Eosine; IHC= Immunohistochemistry; UC= Umbilical Cord.
